# Ultrastructure of Antennal Sensilla in Adults of *Dioryctria rubella* Hampson (Lepidoptera: Pyralidae)

**DOI:** 10.3390/insects12090821

**Published:** 2021-09-14

**Authors:** Jin Xu, Caiping Deng, Wenfeng Lu, Sanan Wu

**Affiliations:** 1 The Key Laboratory for Silviculture and Conservation of Ministry of Education, Beijing Forestry University, Beijing 100083, China; geruibiyuan@126.com; 2 College of Forestry, Shanxi Agricultural University, Jinzhong 030801, China; forestdeng99@126.com; 3 Forest Protection Station of Daxing District of Beijing City, Beijing 120600, China; yljlbz@bjdx.gov.cn

**Keywords:** *Dioryctria rubella*, antenna, sensillum, scanning electron microscopy

## Abstract

**Simple Summary:**

The pine shoot moth (*Dioryctria rubella*) is a major pest on pine trees in China. It damages the branches and cones of the trees. However, little is known about this pine pest. Identifying the olfactory receptors on its antennae is critical for controlling the moth. Therefore, we studied the different types of sensilla present on the antennae of the pine shoot moth and their morphology using scanning electron microscopy. Results showed that the antennae of the moth were filiform. This form of antennae is common and can be divided into three parts: a basal scape, a pedicel, and a flagellum consisting of flagellomeres. As the sensilla play a vital role in the control of this pest, the research presented is a thorough inventory of sensilla on the antennae of the pine shoot moth. This information is important for further functional studies of the antennae of this pine pest.

**Abstract:**

Antennal sensilla play an essential role in insect life because they receive environmental cues. *Dioryctria rubella* is an important pine pest in China, but information on the morphology and distribution of its sensilla is limited. To elucidate the mechanism of insect-plant chemical communication, we examined the insect antennae and sensilla by scanning electron microscopy. The results showed that the antennae of *D. rubella* were filiform and consisted of a basal scape, a pedicel, and a flagellum with tapered flagellomeres. We identified seven types of sensilla, including trichodea, coeloconica, auricillica, basiconica, styloconica (two subtypes), Böhm’s bristles, and squamiformia, all of which were distributed on the antennae of both sexes. Nevertheless, some sensilla exhibited various degrees of sexual dimorphism; for instance, sensilla trichodea, squamiformia, and basiconica were more abundant in males than in females. Many pores were observed on the surface of the cuticular wall in sensilla trichodea and auricillica, and their biological function may be related to olfaction. This study presented a thorough inventory of sensilla on the antennae of *D. rubella* and laid a solid foundation for future functional studies.

## 1. Introduction

Insect antennae play a crucial role in acquiring mates and food, searching for spawning sites, and escaping adverse environmental conditions [1]. The surface of the antennae is equipped with a wide variety of sensilla that vary in structure and function. Sensilla receive complex environmental cues, including chemical signals from mates, host plants, and predators [2,3]. To understand the olfactory behaviors and recognition mechanisms of insects, it is necessary to identify the types, distributions, and functions of antennal sensilla and to study the morphology and structure of olfactory receptors.

*Dioryctria rubella* Hampson (Lepidoptera, Pyralididae, Phycitinae, Dioryctria) is a major pine pest in China [4]. It is distributed throughout more than 20 provinces, autonomous regions, and municipalities in China where it seriously affects the normal growth and shape of the *Pinus massoniana* [5,6,7]. The larvae infest shoots and branches of young Masson pine plants, leading to irregular tunnels in branches, broken shoots, clustered side shoots, and deformed broom-like crowns. *D. rubella* is particularly difficult to control because of its covert behavior [6]. However, little is known about the morphology and distribution of antennal sensilla in *D. rubella*, or their role in basic biological and ecological functions. This has greatly restricted the development and application of an insect olfactory modulation model.

In this study, we conducted a morphological examination on the type and distribution of sensilla on the antennae of *D. rubella*, elucidated differences between male and female adults, and discussed their potential functions.

## 2. Materials and Methods

### 2.1. Insects

Damaged pine branches were collected from an experimental nursery in Wanggezhuang Village, Daxing District, Beijing and cleaned with water. Redundant pine branches were removed, and cuts of damaged branches were covered with a cloth. Water was sprayed on the surface of the cloth every day to maintain humidity. After pupation of larvae, branch tips were cut open, and the pupae were placed in a feeding box with a filter paper-lined at the bottom. A small amount of water was sprayed on the filter paper regularly every day until the adults emerged. At 1–2 d post-emergence, fresh female and male *D. rubella* adults were used as the source for imaging.

### 2.2. Scanning Electron Microscopy (SEM)

Five female moths and five male moths were selected for SEM observations. The antennae were quickly cut off from the head with a scalpel under a dissecting microscope (Olympus, szx-16, Tokyo, Japan), washed repeatedly with distilled water, and immersed in a 70% ethanol solution containing a small amount of Triton X-100. The specimens were refrigerated overnight at 4 °C. The next day, the antennae were cleaned in an ultrasonic cleaner for 10 s, and dehydrated through a graded series of 80%, 90%, 95%, 100% ethanol: water; antennae were maintained for 15 min at each gradation. After critical point drying with CO_2_, the antennae were mounted on SEM sample holders using double-sided adhesive tape, sputter-coated with gold, and examined using an S-3400N scanning electron microscope (Hitachi, Tokyo, Japan) at 3 kV and 10 kV.

### 2.3. Terminology

The antennal sensilla of *D. rubella* adults were classified and named based on the external morphology, surface features, and presence or absence of pores of sensilla. To avoid inconsistency in terminology, we have followed the nomenclature from preceding studies [8].

### 2.4. Measurements and Statistical Analysis

The length and basal diameter of antennal sensilla were measured using ImageJ (National Institutes of Health, Bethesda, MD, USA). Statistical analysis of male and female differences was conducted using the Mann-Whitney U test in SPSS 25 (IBM, Armonk, NY, USA), and all data are presented as mean ± standard error. Image processing was performed using Photoshop 7.0 (Adobe, San Jose, CA, USA).

## 3. Results

### 3.1. General Morphology of Antennal Sensilla in D. rubella Adults

**Figure 1:**
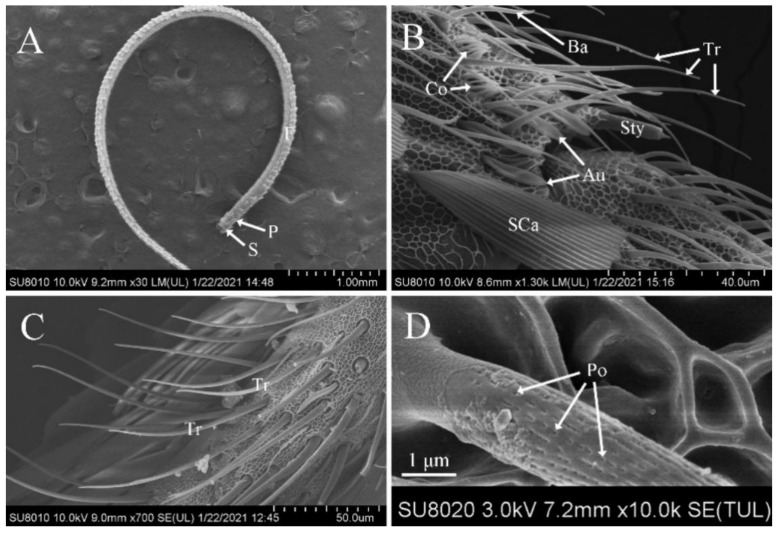
Scanning electron micrographs of the antennae and trichodea sensilla in *Dioryctria rubella*. (**A**) General morphology of antennae; (**B**) various sensilla on the antennae; (**C**) sensilla trichodea (Tr); (**D**) close-up of sensilla trichodea. Au, sensilla auricillica; Ba, sensilla basiconica; Co, sensilla coeloconica; F, flagellum; P, pedicel; Po, pore; S, scape.

The antennae of *D. rubella* adults were filiform and consisted of a scape, a pedicel, and a flagellum. The scape and pedicel consisted of one article each, whereas the flagellum was composed of distally tapered flagellomeres. The dorsal surface of each flagellomere of the flagellum was covered by layered scales. Most sensilla were distributed on the ventral and lateral surfaces of the antennae (Figure 1A).

The length of the antennae was significantly different between males (6774.20 ± 193.27 μm) and females (6345.24 ± 131.38 μm). The scape in the males (length, 329.64 ± 32.17; width, 131.97 ± 14.54 μm) was significantly larger than that in females (length, 281.41 ± 29.74 μm; width, 112.6 ± 15.83 μm). Similarly, the pedicel in the males (length, 63.69 ± 4.35 μm; width, 54.22 ± 4.51 μm) was also significantly larger than that in females (length, 53.39 ± 3.24 μm; width, 49.63 ± 3.23 μm). The number of flagellomeres was similar in males and females; however, the flagellum was significantly longer in males than in females (Table 1). Based on the external morphology of sensilla, seven different types were identified: trichodea, coeloconica, auricillica, basiconica, styloconica (two subtypes), Böhm’s bristles, and squamiformia.

### 3.2. Features of Sensilla Trichodea

Among all the antennal sensilla in *D. rubella*, sensilla trichodea were the most widespread and numerous on the antennae of males and females. Most sensilla trichodea were found on the ventral and lateral sides of the flagellum, and fewer were observed on the dorsal surface. They had a hair-like shape with a thick base and a slender shaft. Most inclined forward on the antenna surface and curved towards the distal tip, almost parallel to the antennae (Figure 1B,C). Moreover, many pores were observed on the surface of sensilla trichodea (Figure 1D). The size and number of sensilla trichodea showed sexual dimorphism; the size in males (length, 51.04 ± 13.49 μm; width, 2.75 ± 0.44 μm) was bigger than that in females (length, 46.45 ± 5.93 μm; width, 2.50 ± 0.52 μm). Additionally, the number of sensilla trichodea was significantly larger in males (2980.6 ± 360.78) than in females (1821.3 ± 162.50) (Table 2 and Table 3).

### 3.3. Features of Sensilla Coeloconica

**Figure 2:**
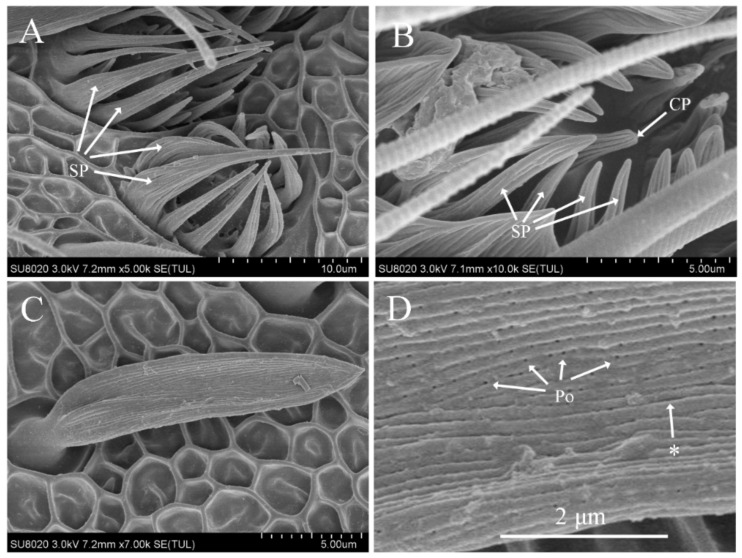
Scanning electron micrographs of sensilla coeloconica and auricillica on the antennae of *Dioryctria rubella*. (**A**) Sensilla coeloconica; (**B**) close-up of sensilla coeloconica; (**C**) sensilla auricillica; (**D**) close-up of sensilla auricillica. Arrow with the asterisk indicates the longitudinal grooves on the sensillum surface. CP, central peg; SP, spine-like protrusions; Po, pore.

Sensilla coeloconica were shallow circular cavities formed by a depression of the antennal cuticle with a protruding sensory peg in the center. The peg had a blunt tip and longitudinal ridges on the surface. The edge of the circular cavity was surrounded by 11–17 finger-like, slightly curved spines of different lengths. Some spines were longer than the sensory peg, and some protruded from the surface of the antennae. The spines curved inward and exhibited longitudinal grooves on their surfaces (Figure 2A,B). Sensilla coeloconica were distributed on the antennae of both males and females, primarily located on the ventral and lateral surfaces, either individually or in clusters. The number was larger at distal flagellomere, whereas none were found on the scape, pedical, or some proximal flagellomere. The length was similar in males (8.85 ± 0.57 μm) and females (9.34 ± 0.89 μm), However, the number showed sexual dimorphism since it was significantly higher in males (287 ± 29.83) than in females (249.4 ± 16.58) (Table 2 and Table 3).

### 3.4. Features of Sensilla Auricillica

The blunt-tipped shaft of sensilla auricillica curved forward and was almost parallel to the antennal surface (Figure 2C). Dense longitudinal grooves were penetrated by many pores (Figure 2D). Sensilla auricillica were distributed on the antennae of both males and females in large quantities, mainly located on the ventral and lateral surfaces. The size in males (length, 17.06 ± 2.94 μm; width, 3.80 ± 0.77 μm) was similar to that in females (length, 17.43 ± 2.95 μm; width, 2.97 ± 0.51 μm) but the number exhibited sexual dimorphism, being significantly higher in males (1128 ± 98.45) than in females (872 ± 79.86) (Table 2 and Table 3).

### 3.5. Features of Sensilla Basiconica

**Figure 3:**
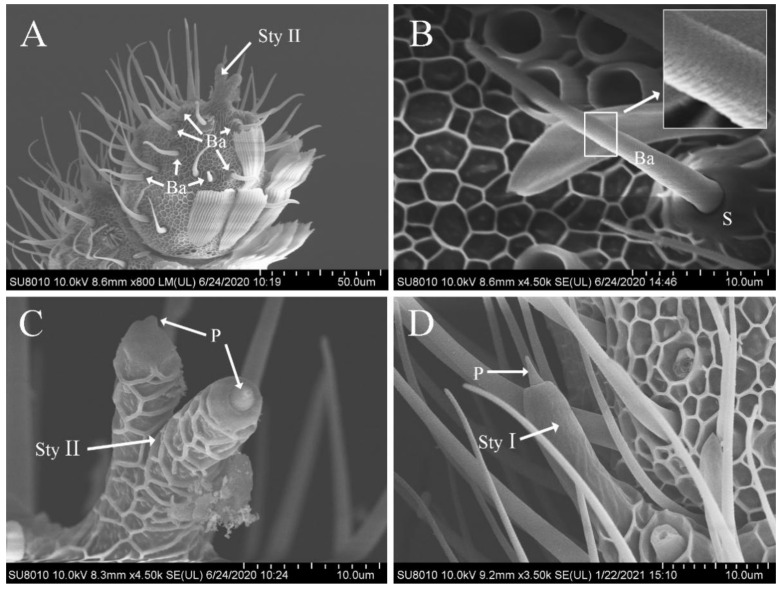
Scanning electron photomicrographs of sensilla basiconica and sensilla styloconica on the antennae of *Dioryctria rubella*. (**A**) Sensilla basiconica and sensilla styloconica (Sty II) on terminal antennae; (**B**) sensilla basiconica (boxes show partial enlargement); (**C**) sensilla styloconica II; (**D**) sensilla styloconica Ⅰ. S, socket; P, peg projection.

Sensilla basiconica were similar to sensilla trichodea in appearance, but did not bend, had a wide base, and were borne on a truncated cone-shaped or conical-shaped raised socket. The blunt tipped shaft inclined forward on the antenna surface and toward the distal tip, with dense diagonal grooves on the surface (Figure 3A,B). Sensilla basiconica were distributed on the antennae of both males and females, sparsely located on the ventral, lateral, and dorsal surfaces. Their number was larger in the distal portion than in the proximal one (Figure 3A), but none were found at the antennal base. The size in males (length, 30.89 ± 3.12 μm; width, 2.23 ± 0.26 μm) was significantly smaller than that in females (length, 34.10 ± 4.42 μm; width, 2.91 ± 0.46 μm), whereas the number did not show any sexual dimorphism (males, 216.0 ± 23.08; females, 194.2 ± 28.14) (Table 2 and Table 3).

### 3.6. Features of Sensilla Styloconica

Sensilla styloconica were stout and thumb-like with an inwardly recessed tip and a small peg projecting from the socket (Figure 3C,D). They were divided into two subtypes (I and II) based on their location and shape and were distributed on the antennae of both males and females. However, none were observed on the pedicel and scape.

The surface of subtype I was smooth, with a cavity at the top and a small peg projecting from the inside (Figure 3D). In most flagellomeres of the antennal flagellum, only one sensillum was observed, slightly inclined forward on the lateral side close to the end of the internode junction and regularly arranged in a vertical direction. The size in males (length, 19.69 ± 1.99 μm; width, 3.91 ± 0.50 μm) was similar to that in females (length, 19.58 ± 1.60 μm; width, 4.12 ± 0.34 μm) (Table 2).

Only one sensillum of subtype II was found on each antenna, situated at the apex of the terminal flagellomere and branched off from the middle part into two similar thumb-like structures with grid-like ridges on the surface and semi-circular projections protruding from the socket at the apex (Figure 3C).

### 3.7. Features of Böhm’s Bristles

**Figure 4:**
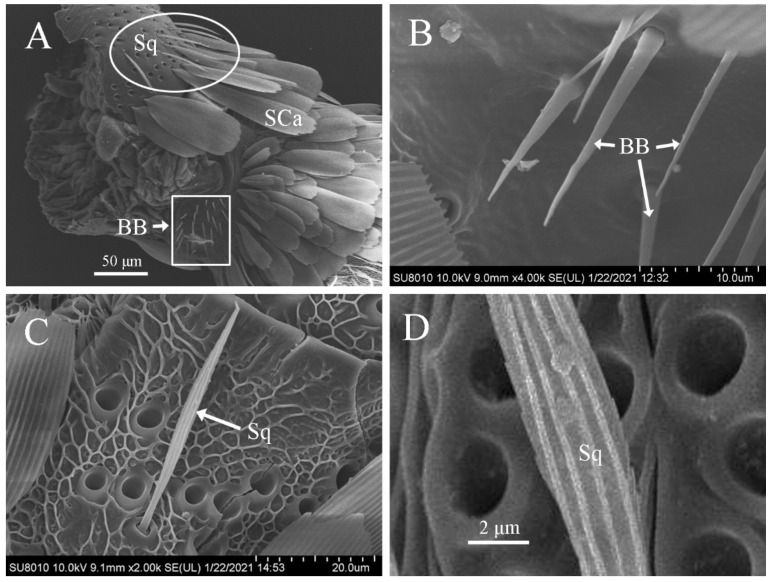
Scanning electron micrographs of Böhm’s bristles and sensilla squamiformia on the antennae of *Dioryctria rubella*. (**A**) Böhm’s bristles (BB) and sensilla squamiformia (Sq) on the basal antennae; (**B**) Böhm’s bristles; (**C**) sensilla squamiformia; (**D**) close-up of sensilla auricillica. SCa, scales socket.

Böhm’s bristles were thin spines with a smooth surface (Figure 4A,B). They were distributed in clusters on the scape and pedicel of the antennae of both males and females, but none were present on the flagellum (Figure 4A). The size in males (length, 12.06 ± 1.64 μm; width, 1.41 ± 0.16 μm) was similar to those in females (length, 11.19 ± 1.77 μm; width, 1.56 ± 0.17 μm (Table 2).

### 3.8. Features of Sensilla Squamiformia

Sensilla squamiformia were in the shape of a flat and thin willow leaf, narrow at the end and wide in the middle with small and narrow scales. The base was set deeply in a cotyloid cavity, and the cuticle had longitudinally arranged grooves (Figure 4A,C). They were primarily distributed on the scape, pedicel, and proximal flagellomeres of antennal flagellum of both males and females. Sensilla squamiformia on the scape and pedicel were distributed in clusters (Figure 4A) but scattered sporadically on the flagellum. The size was similar in males (length, 24.41 ± 2.05 μm; width, 2.32 ± 0.25 μm) and females (length, 25.82 ± 3.10 μm; width, 2.47 ± 0.26 μm) (Table 2).

## 4. Discussion

Insect antennae play a pivotal role in fundamental biological behaviors such as foraging, courting, reproducing, and hiding from natural enemies. Different types of sensilla on the antennae can sense external environmental stimuli. Clarifying the types, distributions, and functions of sensilla is of great significance for understanding the adaptive interrelationship of insects with the environment, as well as their behavior [3].

In the present study, we studied the external structure and distribution of sensilla on the antennae of *D. rubella* using SEM. As with other lepidopteran insects, seven different types of sensilla were identified on the antennae of *D. rubella* adults, including trichodea, coeloconica, auricillica, basiconica, styloconica (two subtypes), Böhm’s bristles, and squamiformia.

Sensilla trichodea are the most important olfactory receptors in lepidopteran insects, and their functions have been extensively studied using molecular biology, electrophysiology, and anatomy techniques. It is known that they play an essential role in two vital activities: courtship and foraging. Previous studies showed that proteins and receptors of sex pheromones were expressed in sensilla trichodea using immunohistochemistry and in situ hybridization [9,10,11,12]. In addition, sensilla trichodea were found to be used for plant odor detection in some lepidopteran insects [13,14,15,16]. In *D. rubella*, they were the most widespread and numerous antennal sensilla, and the sensilla have pore structure on the wall, which represents a chemical sensing function. However, more research is needed to verify their biological functions using molecular biology and electrophysiology techniques.

Sensilla coeloconica on the antennae of *D. rubella* are structurally similar to those of other lepidopteran insects such as the dogwood borer [17] and *P. xylostella* [18]. Yan et al. (2017) [18] used transmission electron microscopy to observe the internal structure of sensilla coeloconica on the antennae of *P. xylostella* and found that dendrites were located inside the central sensory peg. However, no dendrites were observed in the finger-like spines surrounding the circular cavity, suggesting that the sensory peg has a sensory function, whereas the finger-like spines may protect it from mechanical damage, desiccation, and moisture [19,20]. Sensilla coeloconica are probably involved in recognizing volatile odor molecules [21,22] and sensing water vapor, carbon dioxide, and changes in humidity [23,24]. Based on the quantity and distribution characteristics, we assumed that sensilla coeloconica in *D. rubella* might also be thermo- and hygro-sensitive.

Sensilla auricillica have been reported to receive plant volatiles in *Scoliopteryx libatrix* [25] and *Cydia nigricana* [26], but they are also thought to detect sex pheromones emitted by female moths [27,28]. In *D. rubella*, sensilla auricillica were highly abundant with pores on the surface, suggesting that these sensilla might be related to olfaction.

Sensilla basiconica are believed to sense sex pheromones [29] and plant volatiles [30,31]. It has been reported that SlituOR12, an odorant receptor, is expressed in the sensilla basiconica of the common cutworm and exclusively tuned to the important plant volatile cis-3-hexenyl acetate [32].

In *D. rubella*, sensilla styloconica were divided into two subtypes based on their morphological features. Subtype I sensillum was larger than subtype II and shared similar morphology and distribution characteristics with those of other lepidopteran insects [17]. Only one sensillum of subtype II was found at the apex of the terminal flagellomere of the antenna, and its structural features and distribution were the same as those on the antennae of *Z. dixolophella* [33]. In *Bombyx mori*, sensilla styloconica are considered responsible for sensing changes in external temperature and humidity [34], whereas they function as chemoreceptors in *C. nigricana* [26]. Sensilla styloconica in *D.* *rubella* might be thermo- and hygro-sensitive.

Böhm’s bristles on the antennae of *D. rubella* had a smooth surface free of pores and were distributed in clusters on the scape and pedicel, similar to those observed in *H. nebulella* [35], *P. xylostella* [18], and other lepidopteran insects. Böhm’s bristles probably do not have any olfactory function [12,13], but they sense mechanical movements and perceive the position and movement of the antennae [8,36].

In *D.* *rubella*, sensilla squamiformia were also distributed on the scape, pedicel, and the first few flagellomeres, similar to those in *H. nebulella* [34] and *C. obducta* [37]. Previous studies revealed that they are probably responsible for sensing external mechanical movements [8,38].

## 5. Conclusions

We observed sexual dimorphism in the type, number, and distribution of sensilla in *D. rubella*. The number and size of sensilla trichodea and basiconica exhibited sexual dimorphism. Sensilla trichodea might be associated with the detection of sexual pheromones and/or host plant volatiles. Overall, our data laid a solid foundation for future functional studies in *D. rubella* using molecular biology, electrophysiology, and anatomy techniques.

## Figures and Tables

**Table 1:** General antennal characteristics in *Dioryctria rubella* adults.

Sex	Antennae Length (μm)	Number of Flagellum Flagellomeres	Scape Length (μm)	Scape Width (μm)	Pedicel Length (μm)	Pedicel Width (μm)	Flagellum Length (μm)
Male	6774.20 ± 193.27 a	62 ± 0.32 a	329.64 ± 32.17 a	131.97 ± 14.54 a	63.69 ± 4.35 a	54.22 ± 4.51 a	6,419.35 ± 129.24 a
Female	6345.24 ± 131.38 b	61 ± 0.40 a	281.41 ± 29.74 b	112.6 ± 15.83 b	53.39 ± 3.24 b	49.63 ± 3.23 b	6,145.23 ± 186.14 b

All data are presented as mean ± standard error. Means with the same lower-case letter in the same column do not differ significantly at *p* = 0.05. *N* = 10 antennae from each sex.

**Table 2:** Size of different antennal sensillum types in *Dioryctria rubella* adults obtained from SEM images.

Types of Sensilla	Length (μm)		Width (μm)	
	Male	Female	Male	Female
Trichodea	51.04 ± 13.49 a	46.45 ± 5.93 b	2.75 ± 0.44 a	2.50 ± 0.52 a
Coeloconica	8.85 ± 0.57 a	9.34 ± 0.89 a	--	--
Auricillica	17.06 ± 2.94 a	17.43 ± 2.95 a	3.80 ± 0.77 a	2.97 ± 0.51 a
Basiconica	30.89 ± 3.12 b	35.10 ± 4.42 a	2.23 ± 0.26 b	2.91 ± 0.46 a
StyloconicaⅠ	19.69 ± 1.99 a	19.58 ± 1.60 a	3.91 ± 0.50 a	4.12 ± 0.34 a
StyloconicaⅡ	--	--	--	--
Böhm′s bristles	12.06 ± 1.64 a	11.19 ± 1.77 a	1.41 ± 0.16 a	1.56 ± 0.17 a
Squamiformia	24.41 ± 2.05 a	25.82 ± 3.10 b	2.32 ± 0.25 a	2.47 ± 0.26 b

All data are presented as mean ± standard error. Means with the same lower-case letter in the same row for “Length” and “Width” do not differ significantly at *p* = 0.05. *N* = 10 antennae from each sex. --, present but not counted.

**Table 3:** Estimated number of antennal sensilla in *D. rubella* adults.

Types of Sensilla	Male	Female
Trichodea	2980.6 ± 360.78 a	1821.3 ± 162.50 b
Coeloconica	287.0 ± 29.83 a	249.4 ± 16.58 b
Auricillica	1128.0 ± 98.45 a	872 ± 79.86 b
Basiconica	216.0 ± 23.08 a	194.2 ± 28.14 a
StyloconicaⅠ	--	--
StyloconicaⅡ	--	--
Böhm’s bristles	--	--
Squamiformia	--	--

All data are presented as mean ± standard error. Means with the same lower-case letter in the same row do not differ significantly at *p* = 0.05. *N* = 10 antennae from each sex, --, present but not counted.

## Data Availability

The data presented in this study are available on request from the corresponding author.
